# A reduced-order closed-loop hybrid dynamic model for design and development of lower limb prostheses

**DOI:** 10.1017/wtc.2023.6

**Published:** 2023-04-04

**Authors:** Josephus J.M. Driessen, Matteo Laffranchi, Lorenzo De Michieli

**Affiliations:** Rehab Technologies Lab, Istituto Italiano di Tecnologia (IIT), Genoa, Italy

**Keywords:** dynamics, simulation, lower limb prostheses

## Abstract

This manuscript presents a simplified dynamic human-prosthesis model and simulation framework for the purpose of designing and developing lower limb prosthesis hardware and controllers. The objective was to provide an offline design tool to verify the closed-loop behavior of the prosthesis with the human, in order to avoid relying solely on limiting kinematic and kinetic reference trajectories of (able-bodied) subjects and associated static or inverse dynamic analyses, while not having to resort to complete neuromusculoskeletal models of the human that require extensive optimizations to run. The presented approach employs a reduced-order model that includes only the prosthetic limb and trunk in a multi-body dynamic model. External forces are applied to the trunk during stance phase of the intact leg to represent its presence. Walking is realized by employing the well-known spring-loaded inverted pendulum model, which is shown to generate realistic dynamics on the prosthesis while maintaining a stable and modifiable gait. This simple approach is inspired from the rationale that the human is adaptive, and from the desire to facilitate modifications or inclusions of additional user actions. The presented framework is validated with two use cases, featuring a commercial and research knee prosthesis in combination with a passive ankle prosthesis, performing a continuous sequence of standing still, walking at different velocities and stopping.

## 1. Introduction

Design and development of lower limb prostheses is impaired by the impossibility to holistically test the prosthesis with end users early in the development process, and by the difficulty of accurately simulating them in a closed loop with the human. The technical design and development of lower limb prostheses is typically based on static or inverse dynamic analyses of existing kinematic and kinetic reference trajectories of (able-bodied) subjects (Fite et al., 2007; Sup et al., 2007; Vallery et al., 2011; Lawson and Goldfarb, 2014; Rouse et al., 2014; Grimmer et al., 2016). Whereas such analyses can provide an acceptable starting point to obtain ballpark figures for the prosthesis’ electromechanical design, they are problematic for subsequent stages of design. A core problem is that the analyses place strong assumptions on human behavior, even though human behavior is highly adaptive. In effect, they do not allow for developments and validations that regard or require closed-loop performance with the human, including controller design. Per evidence, recorded trajectories from amputees wearing their state-of-the-art prosthesis have been found to differ significantly from each other and that of non-amputees (Wühr et al., 2007; Grimmer and Seyfarth, 2014; Bisbee III et al., 2016). Deviations are partly caused by differences in the dynamical system (e.g., different mass and stiffness of materials, purely revolute versus polycentric joints Pfeifer et al., 2012, etc.), but also by psychological factors, for example, causing some to (inadvertently) keep the knee extended throughout stance (Bisbee III et al., 2016). In essence, humans are not bound to realize only the most economic trajectories. Instead, they are rather unconstrained in their actions, and free to deviate from their default gait, to which the hardware and its controller have to act appropriately as well.

If developments and validations could only proceed with a trial-and-error-based process that includes the real hardware and humans in the loop, then the design life cycle is prone to be costly and lengthy. Especially in the field of medical robotics, experimental trials follow late in the design stage, as they require the hardware to be already fully built, commissioned, safe and certified. The discovery of a design flaw this late in the design stage is expensive. In addition, experimental trials are plagued by safety protocols, hardware repair and real-time constraints, further slowing progress down.

The adaptive and broad repertoire of human behaviors calls for a holistic modeling approach that includes both the human and prosthesis in the loop, to close the gap between ballpark calculations that are carried out at the beginning of the design cycle and experimental trials that can only be carried out at the very end of the design cycle. The challenge is to produce a comprehensible model that can reproduce sufficiently realistic human behavior for the prosthesis to perform closed-loop dynamic analyses.

Many neuromusculoskeletal models have been developed and employed in order to understand and predict human gait (Anderson and Pandy, 2001; Paul et al., 2005; Hicks et al., 2015). Such models have previously been used to fine-tune a prosthesis design (Fey et al., 2012; Silverman and Neptune, 2012; Handford and Srinivasan, 2016) for an optimal performance of the human, such as for reduction of metabolic cost or fatigue (Ackermann and van den Bogert, 2010). However, most of these studies focus on a single gait pattern and do not incorporate other actions, possible deviations, or disturbances. They are assumptive about human behavior and do not analyse forward-dynamic stability, for which they are not usable for studies that incorporate closed-loop control. Few studies have focused on realizing forward-dynamic self-stabilizing gait by encoding human control principles and muscle reflexes (Gerritsen et al., 1998; Geyer and Herr, 2010). The model of Geyer and Herr (2010) can deal with disturbances (e.g., slopes, steps) and has been extended to adapt different walking speeds (Song and Geyer, 2012). OpenSim is another popular and open-source platform that models the human neuromusculoskeletal complex dynamically (Delp et al., 2007), which has since been extended to allow for forward-dynamic studies on self-stabilized gait and for the inclusion of a prosthesis (Geijtenbeek, 2019). However, hand-tuning is an unfeasible tasks for such models; optimizations are necessary, with the typical objective of minimizing metabolic cost or joint contacts. In general, optimization problems are difficult to define in the early stages of a design project, they provide lack of insight into the system, and they are time consuming. Popular optimization methods that can incorporate closed-loop control include shooting methods (Geijtenbeek, 2019) and reinforcement learning (Kidziński et al., 2018). As a result of the multidimensional complexity of the system, such optimizations last hours to days, or days to weeks, for shooting methods and reinforcement learning, respectively. This does not yet take into account co-optimization of the prosthesis hardware and/or controller, which further increases the dimensionality of the problem. In addition, the optimization problems are typically focused on limited human target behavior (e.g., walking). Extending that behavior to incorporate multiple actions or change of human intent is not straightforward, which reduce their appeal for developing high level control on intent detection.

In contrast, studies that make use of simpler dynamical models that do not model the whole human, its muscles and control principles, frequently segment and tailor them according to specific phases, such as swing, stance and gait initiation (van Keeken, 2003; Pagel et al., 2017). Whereas these models can be useful to explain the mechanics of their respective phase, the discontinuous nature of such models provide limited usability to holistic studies that aim to incorporate closed-loop controller behavior, and they have to deal with corresponding initial and terminal conditions.

The objective is to create a minimally simplistic forward dynamics simulation framework of the human-prosthesis system that produces realistic dynamics of possible cyclic and non-cyclic human activities on the lower limb prosthesis, for the general purpose of prosthesis hardware and controller design and validation. It is important that motions and forces imposed on the prosthesis are within realistic bounds and representative of a possible human action, but less priority is given to the correct modeling of the human muscles. The framework is not designed to address a specific prosthesis design or specific user needs. Since anyway the final behavior of the human is uncertain, the focus is not on what the human does *ideally*, but on what it *could* do, in order to design and validate the prosthesis and its controller for these behaviors. Toward this purpose, the model should be sufficiently simple and comprehensible to the extent that hand-tuning remains a possibility to achieve different but stabilizable and realistic behaviors. It should also be possible to change behavior during a single continuous simulation, in order to enable the design and validation of high level control such as intent detection. Lastly, simulations ideally take on the order of seconds instead of hours.

Under the pretext that the human is complex and remains in control, we do not attempt to recreate its controller, but aim to replicate only that what is necessary to obtain and maintain realistic dynamics of the residual leg. The presented approach is inspired from the field of legged robots, where reduced-order models have shown to accurately capture the most relevant dynamic behavior of the otherwise complex walking machines (McGeer, 1990; Goswami et al., 1997; Geyer et al., 2006). In this work, the human too is modelled as a reduced-order system, consisting of only the residual leg (containing the prosthesis) and the human trunk as rigid bodies in a multi-rigid-body system. This system is kept upright through external forces on the trunk, to represent the presence of the intact leg and other limbs. For walking in particular, realistic forces are generated by employing the spring-loaded inverted pendulum (SLIP) model to represent the intact leg, as it is well known for being able to encode principles of walking dynamics, despite its simplicity (Geyer et al., 2006; Blickhan et al., 2007).

## 2. Framework overview

**Figure 1.**
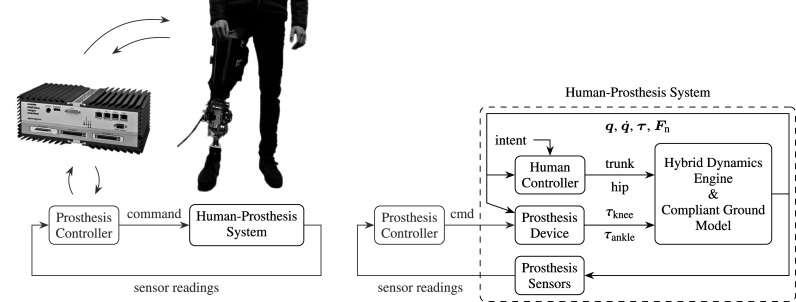
Left: highest level abstraction of the simulation framework, consisting of the Prosthesis Controller and Human-Prosthesis System, running in discrete time (colored) and continuous time (black), respectively. The modelled Human-Prosthesis System can be replaced for a real human and prosthesis to perform hardware-in-the-loop experiments, for example, by utilizing a CAN interface or a real-time target machine such as Speedgoat (Speedgoat, 2020; Guercini et al., 2022). Right: main modules of the modelled Human-Prosthesis System. The human controller can generate either forward (torques) or inverse dynamics (positions, velocities and accelerations) inputs of the trunk and hip for the hybrid dynamics engine.

A high level overview of the framework is depicted in Figure 1. At its highest level (shown at the left in the figure), it consists of the knee and/or ankle *prosthesis controller*, to represent its microprocessor(s), and the *human-prosthesis* *system*, which represents the physical prosthesis and the human wearing it. Effectively, the human-prosthesis system runs in continuous time, implying that the use of variable integration time steps is permitted, whereas the prosthesis controller runs in discrete time (depicted colored in Figure 1), with a fixed time step that corresponds to the controller frequency of the microprocessor. This distinction facilitates a possible adaptation to a rapid control prototyping setup, where the human-prosthesis system can be replaced by the real human and prosthesis, and the prosthesis controller by its implementation on the prosthesis’ microprocessor or real-time target machine such as a Speedgoat (Pagel et al., 2017; Speedgoat, 2020), which strictly runs in discrete time. The prosthesis controller and each of the modules of the human-prosthesis system (as shown at the right in Figure 1) are introduced below.

The *prosthesis controller* module receives sensor readings, such as those from joint encoders, force sensors and an inertial measurement unit, and sends command signals to the prosthesis device, such as desired motor position or torque. For reasons of practicality and efficiency, controllers of the lowest hierarchical level that run at high frequencies (typically 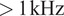 ) and on dedicated control boards—such as motor servo controllers that generate pulse-width modulated motor voltage—can be ignored or simplified and embedded in the prosthesis device, to be treated part of the continuous time system.

The *prosthesis device* module calculates the actual knee torque 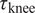 and ankle torque 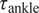 from the commands, and possible models of friction, kinematics (e.g., a linkage model if the knee is actuated with a linear actuator and a lever), and motors or variable dampers and their simplified servo controllers. The torques are then applied to their respective joints of the multi-rigid-body mechanism that models the residual leg and trunk, which is fully described in Section 3. Both the prosthesis controller and device are case-specific; two case studies are provided in Section 5.

The *human controller* module takes user intent as input and generates a combination of joint torques and position trajectories for the hip and trunk that are applied to the multi-rigid-body model. Forces or trajectories applied to the trunk represent those caused by the presence of an intact leg and arms in reality, as they are not explicitly included as rigid bodies in the model. The human controller is elaborated in Section 4.

The *hybrid-dynamics engine* module performs both forward- and inverse-dynamics calculations for this model. It takes inputs from both the prosthesis controller and human controller. The hybrid-dynamics engine interacts with the *compliant ground model*, which generates external forces that act on the multi-rigid-body mechanism. The two are further elaborated in Section 3.

Lastly, the *prosthesis sensors* module takes various outputs from the dynamic model and prepares them as sensor readings for the prosthesis controller. This includes at least a step of temporal discretization, but could also include a quantization to realize finite encoder resolutions and can be used to introduce (random) noise.

## 3. Dynamic modeling

**Figure 2.**
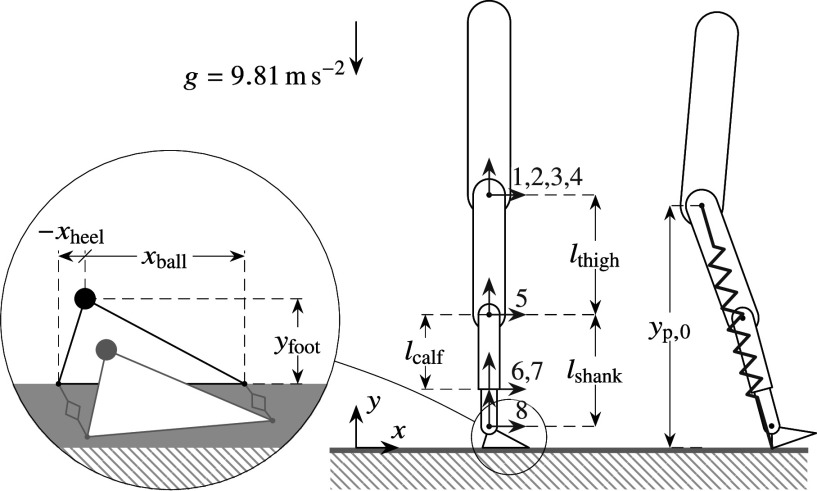
Leg Model: depiction in a shifted zero configuration ( 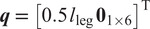 ) (center); positioning of its foot’s two ground contact points (left); and its reference touch down configuration for defining that of the overlaid SLIP model (right).

The residual leg and trunk are modelled as a planar multi-rigid-body mechanism. It consists of a serial chain of eight joints and bodies, so that joint 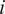 connects body 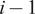 to body 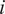 , with body 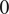 being the ground. The first three joints implement the floating base of the trunk. The next five joints implement hip rotation (4), knee rotation (5), tube sensor extension (6) and rotation (7), and ankle rotation (8), respectively. Joints can be added or removed depending on the application. For example, tube sensor joints are introduced for the purpose of obtaining realistic readings from local force and moment sensors, which are frequently but not necessarily present in prosthesis designs. An additional prismatic joint could be introduced here to obtain shear force readings. Additional joints can also be introduced on the thigh if it is desired to model socket stiffness or to read interactive forces with the amputee, as showcased in Section 5.2.3. The 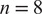 joint positions are elements of the tuple 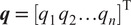 and corresponding joint forces or torques elements of the tuple 
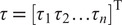
 . A complete model description is provided by Figure 2, which shows joint coordinate frames 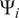 in red, and by Table 1, which lists joints types, kinematic parameters and inertial parameters: body mass 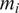 , center of mass coordinates 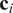 with respect to 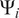 , and inertia 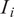 with respect to 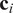 .

**Table 1.** Joint and body data (total mass m = 75kg)

	Joint		Body		*m_i_*	*I_i_*	*c_i_*
*i*	Type^a^	Name	Name		[kg] (%)	[kgm^2^]	[cm]
1	px	Trunk travel	–		0	0	[0, 0]
2	py	Trunk height	–		0	0	[0, 0]
3	r	Trunk rotation	Trunk		62.5 (83.3%)	4.401	[0, 32.5]
4	r	Hip	Thigh		9 (12%)	0.1337	[0, –18]
5	r	Knee	Calf		2.8 (3.7%)	0.03	[−1, –12]
6	py	Tube extension	–	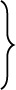 shank	0	0	[0, 0]
7	r	Tube rotation	Tube		0	0	[0, 0]
8	r	Ankle	Foot		0.7 (0.93%)	0.0018	[6.25, –5.33]

*Note.* Parameters choices are based on anatomical data (Plagenhoef et al., 1983; Tilley and Dreyfuss, 1993), corrected for the inclusion of a lower limb prosthesis, which weighs less than a healthy leg. Percentages with respect to the total leg length of 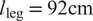 .

a Joint type: revolute (r) or prismatic in their local *x-* (px) or *y-*axis (py).

Body sizing is according to anatomical data of a 50th percentile adult man of the U.S. population (Tilley and Dreyfuss, 1993) (177 cm, 78.4 kg), having a leg length of 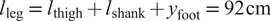 . Proportions of inertial data are based on anatomical data too (Plagenhoef et al., 1983), but corrected for the replacement of one healthy leg for a prosthetic leg, which is typically lighter, so that the total mass of the system (amputated person and prosthesis) is 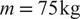 . The mass of the whole upper body and healthy leg are included in the trunk body. Note that, as a result, whereas in a healthy person the mass of the upper body and a leg account for roughly 77% of the total body mass (55.1% and 22.5%, respectively, for males Plagenhoef et al., 1983), here they represent more (83%).

### 3.1. Hybrid-dynamics engine

The hybrid-dynamics engine performs dynamics calculations in which a selection of joints is configured as a forward- and the others as an inverse-dynamics joint (Featherstone, 2008). In case of the former, joint torque 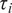 is provided and the hybrid-dynamics engine calculates joint acceleration 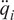 , in which case joint position 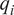 and velocity 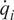 are obtained through numerical integration. In case of the latter, 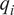 , 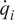 and 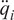 are provided to calculate 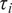 . Joint configurations are as follows:

- Joints 5 and 8 are that of the knee and ankle and controlled by the prosthesis, for which they are forward-dynamics joints ( 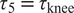 and 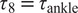 ).
- Joints 6 and 7 are introduced for the purpose of obtaining force and moment readings, for which they are configured as inverse-dynamics joints, and in fact locked, so that 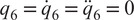 and 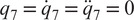 .^1^ 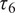 corresponds to vertical force and 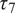 to the bending moment.
- Joints 1 to 4 are configured differently depending on the scope of the simulation. If ground contact is involved (stance), at least two independent joints must be configured as forward dynamic joints to assure motion freedom, typically trunk translation (1 and 2). If also ground impact is involved, like in walking, it is advised to configure also the hip joint as forward dynamic joint in order to reduce impact force and slip. The case studies presented in this manuscript use this configuration.

### 3.2. Symbol conventions

For readability and consistency with literature, additional symbols are introduced to represent variables with units different from the SI system and signs that could be opposite from their definition in the dynamic model in Section 3.1. Angular positions and velocities 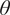 and 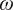 are expressed in  and rpm, respectively. Corresponding rotational joints are defined so that a positive value corresponds to flexion: we have trunk angle 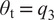 , knee angle 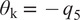 and ankle angle 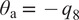 . In addition, hip angle 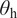 is expressed with relation to the absolute vertical, so that 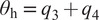 . Forces 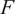 are expressed per body weight (N N^−1^) and moments 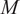 and torques 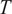 per body mass (N m kg^−1^). Furthermore, several sign changes apply: we have trunk horizontal force 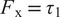 , trunk vertical force 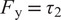 , trunk torque 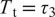 , hip torque 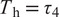 , knee torque 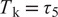 , tube force 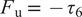 , tube moment 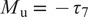 and ankle torque 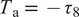 . Values for associated controller parameters are expressed in corresponding units.

### 3.3. Compliant ground model

Ground contact is modelled by a compliant ground model (Azad and Featherstone, 2010), instead of by employing non-continuous impulsive dynamics and switching constraint functions. This approach allows for impact forces to remain realistically finite, and can be used to take an extent of compliance into account that is found in a real foot prosthesis, socket and hip joint, which is otherwise not modelled by the multi-rigid-body mechanism. The compliant ground model implements a non-linear spring damper element between defined contact points and the ground, so that the normal force 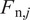 of a point 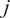 equals

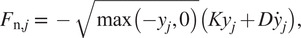

where 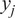 is the absolute y-coordinate of point 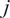 , and 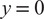 defines the ground plane. At least two contact points must be defined in order to exercise a moment: one in the heel and one in the ball of the foot, respectively, defined by the coordinates 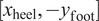 and 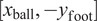 in the coordinate frame of the foot, as shown in Figure 2. Kinematic parameter choices are listed in Table 1. Note that the distance between the heel and ball essentially represents the effective foot length, which is found to be between 0.63 to 0.81 times the total length of a prosthetic foot (Hansen et al., 2004). Stiffness and damping constants for the two contact points are set to 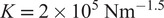 and 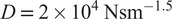 , respectively. This stiffness implies that the foot sinks approximately 1.5 cm in the ground when the total body weight (ca. 750 N) is applied on the foot, which might suggest that the floor is rather soft. However, the stiffness also models vertical or longitudinal compliance found in a real leg and foot prosthesis, which is otherwise not taken account for by the rigid bodies and its rotational joints of the presented leg model. An increased stiffness leads to higher internal peak forces and torques at impact, but was anyway found to not significantly affect system dynamics: increasing it tenfold did not destabilize the system. Alternatively, a higher stiffness can be combined with the tube extension joint (joint 6) configured as a stiff forward dynamics joint, as mentioned in Section 3.1, so that it too acts as a spring. The damping value was tuned heuristically to achieve an approximate critical damping behavior for typical impacts that occur during walking. The model furthermore implements the ability to both stick and slip, with a friction coefficient of 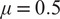 . Corresponding tangential forces are calculated using the same spring-damper constants.

## 4. Human controller

**Figure 3.**
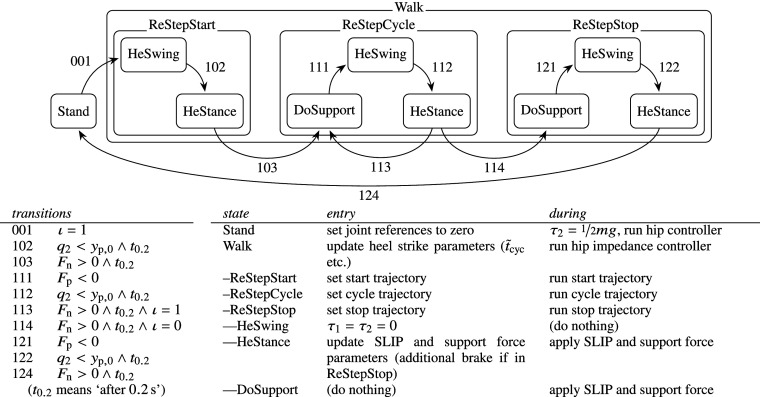
Human controller overview, including a depiction of FSMs and associated control actions (He = healthy/intact leg, Re = residual leg, DoSupport = double support), which implements standing, walking at different ambulation speeds, and transitioning between the two.

The human controller implements the ability to stand still, walk at different speeds, and to initiate and terminate the walking gait. It uses a dedicated finite state machine (FSM), shown in Figure 3, which takes human intent and intentional velocity as input, and regulates the dynamic synthesis of reference trajectories and joint forces. Human intent refers to a desired action type of the user, such as standing or walking, and a switch between these two result in gait initiation or termination.

Trunk translation joints (joints 1 and 2, see Figure 2) are configured as forward-dynamics joints, in order to assure motion freedom and a smooth transition between the various swing and stance phases. Forces are applied to the trunk during the stance phase of the intact leg to represent the presence of the intact leg, which is necessary because the intact leg is not part of the multi-rigid-body model, and the system would otherwise fall down. During walking, it is represented by the SLIP model. The SLIP model can be accompanied by additional forces on the trunk, which in a reality can represent deviating ground contact forces of the intact leg, inertial forces of the human body complex or external disturbances. In particular, since the SLIP model is purely dissipative, an additional forward force is necessary if it is desired to inject energy by the intact leg, and thus to allow for an energetically symmetric gait. Otherwise, the only source of energy injection is that from the residual leg during its stance phase, and in particular only from the hip if the lower limb prosthesis is passive. Such a force can also be used to regulate the intended ambulation velocity 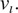 The implementation of the SLIP model and optional additional trunk forces are further explained in Sections 4.2 and 4.3, respectively.

The trunk and hip (joints 3 and 4) are modelled to be stiff and follow time-based position trajectories in combination with an initiation or reset reflex, rather than being dynamically controlled by a balance controller. The rationale for this feed-forward approach is that also humans do not tend to continuously and consciously balance themselves during a single stance phase, and by keeping the assumptions of human behavior simple, the focus remains on developing or testing the performance of the prosthesis controller, rather than on understanding the human’s capability to correct the prosthesis’ control actions. The initiation or reset reflex refers to resetting the time-based trajectory at heel strike of the residual leg, so that the step time is not constrained to a fixed value.

The automated regulation of both the initiation of reference trajectories as well as that of the SLIP model—which together constitute the human controller—were found to significantly improve stability. Stability here refers to the ability to maintain a walking gait, that is, to not eventually (trip and) fall. This suggests the convergence to a limit cycle gait if none of the environmental inputs or human intent (walking intent and intended ambulation speed) change. The employed method to generate smooth trajectories in combination with dynamic resets or change of user action or ambulation velocity is further explained below.

### 4.1. Trajectory generation

Trajectory generation is realized so that smooth trajectories are obtained for applicability as inverse dynamics references, while not being predetermined to allow for interactive change of user intent, ambulation velocity and varying step times. This is achieved by a two-step procedure: rough and smooth joint reference generation.

#### Rough joint references

**Figure 4.**
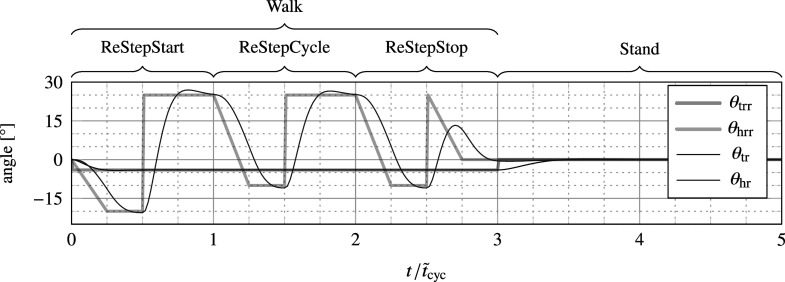
Predefined rough reference trajectories 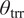 and 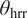 for the trunk and hip, respectively, for each of the four main states of the human controller (see Figure 3), normalized for the anticipated stride cycle duration 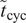 (see *x*-axis). The resulting smooth reference trajectories 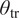 and 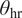 for this particular chain of reference trajectories are depicted as well, as obtained by passing the rough reference trajectories through the filter in equation 1. While walking, the trunk is sent into a forward lean of 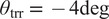 , and the hip roughly follows a trajectory between 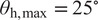 and 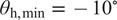 .

Firstly, rough joint position reference templates are predefined for every user action, which can be discontinuous. These include a reference trajectory for standing still (i.e., a constant zero joint angle), an initiation step, a termination step, and a cyclic walking step, the latter of which normalized for step time, as shown in Figure 4. Through the FSM of the human controller, these templates are scaled in time, cut, and stitched together to define the rough joint reference 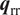 , based on user intent (walking or standing still), change of intent (starting or stopping a walk), intended ambulation speed, and step cycle completion.

If the human intent changes from stand to walk, the human controller will pass once through a transition state to initiate gait (ReStepStart), and vice versa (ReStepStop). In reality, gait initiation and termination can be executed by both legs and in different ways (van Keeken et al., 2008). However, in the presented model, the initiation step is implemented such that the intact leg makes the first step (executed by the SLIP model). Forward thrust is generated with the residual leg by flexing the hip while standing. During walking, the reference position template of the next step is defined at the heel strike (step initiation reflex) of the residual leg (i.e., when transitioning to ReStepCycle or ReStepStop) according to the registered intent and intended ambulation speed at that time instance, at which point the reference trajectory of the previous step is terminated and that of the new step initiated. In particular, the reference trajectory is scaled in time as function of the intended ambulation speed. As such, no different trajectories are defined for different ambulation velocities, though such an implementation would be possible too. The scaling is done with an estimate of the duration of the next step cycle 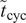 according to 
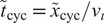
 , where the estimate of stride length 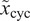 is the stride length of the previous step cycle 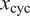 , measured as the distance between the heel of the residual leg at previous and current heel strike (initial estimate 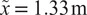 ).

#### Smooth joint references

Secondly, smooth position reference trajectories 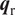 , continuous velocity reference trajectories 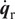 and discontinuous but finite acceleration reference trajectories 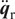 are obtained through a two-dimensional filter in order to make them eligible as inputs for the hybrid dynamics engine for joints configured as inverse-dynamics joints. Because of the filter, the output remains smooth even during state switches of the FSM, which is crucial as state changes are not timed and can occur unexpectedly due to, for example, the step initiation reflex.

The filter to obtain 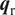 and its derivatives is implemented as follows:
(1)

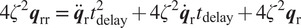

with a damping ratio 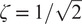 and a variable time delay 
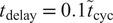
 (during standing, 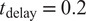 ) so that the shapes of the reference trajectories 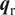 ( 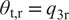 and 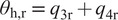 for the trunk and hip) become invariant of the commanded ambulation velocity. The result of the filter output is also shown in Figure 4.

An additional advantage of the two-step procedure for trajectory generation is that it requires the definition of only compact and tangible or descriptive data structures that describe the approximate motion of the leg (i.e., those of the rough reference trajectories), rather than having to rely on long lookup tables that are difficult to customize. As shown in Figure 4, the hip trajectory for a cyclic walking step can be described as gradually moving backwards from a starting flexion angle 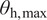 to an extension angle 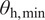 during stance, after which it accelerates back toward its starting angle during the beginning of swing, while anticipating the next foot strike.

#### Joint commands

As mentioned in Section 3.1, both the trunk and hip rotation joints are eligible to be configured as inverse-dynamics joints without over-constraining the body kinematics during stance. This can be realized by directly taking the generated smooth reference trajectory 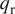 and derivatives of the corresponding joint as inputs to the hybrid-dynamics engine. By doing so for the trunk, the system dynamics are equivalent for assuming the trunk to be a point mass, which has previously shown to be a fair approach in simple dynamic walking models (McGeer, 1990; Geyer et al., 2006; Blickhan et al., 2007). When the trunk is a point mass, it is implied that—as long as no joint torque limits are imposed on the trunk joint, and as long as hip rotation directly compensates for trunk rotation (which it does, because hip rotation trajectories are defined with respect to the absolute vertical, i.e., 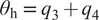 )—the trunk orientation does not affect the outcome of the dynamics. Nevertheless, for purpose of demonstration and aesthetics, the trunk is sent in a forward lean of 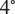 while walking, as shown in Figure 4.

The hip joint follows a more dynamic trajectory. Whereas it too can be configured as inverse-dynamics joint, it is instead configured as a forward-dynamics joint to follow its reference trajectory with a stiff joint-impedance controller, in order to moderate impact forces and to eliminate occurrences of (micro-)slip throughout stance:

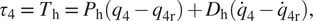

with a manually tuned stiffness gain 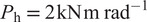 while walking and 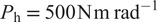 while standing, and damping gain 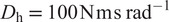 . The stiffness is manually tuned to be maximal for eliminating visible foot slip during stance.

### 4.2. Walking: SLIP trunk forces

During stance of the intact leg (HeStance and DoSupport), the trunk is subject to a force from the SLIP model 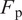 (as displayed also in Figure 2), a forward thrust 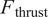 and optionally a vertical lift force 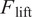 :

The resulting applied trunk forces are calculated as follows:

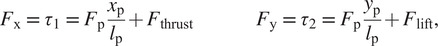

in which 
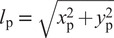
 , 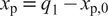 and 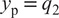 are the pendulum’s actual length and its corresponding *x*- and *y*-components, respectively. 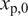 is the pendulum’s floor coordinate. 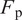 is calculated as 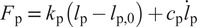 , in which the parameters 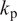 and 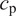 are linear stiffness and damping coefficients, respectively, and 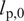 the pendulum’s rest length.

Parameters are chosen so as to reproduce the double ‘camel back’ bounce that is typically observed in the ground reaction force of healthy human walking. Damping 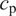 has been added in order to account for natural losses at ground impact and to modulate an excess of bouncy behavior. Accordingly, 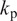 and 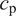 are calculated as follows:

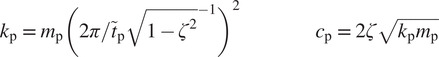

with the estimated pendulum ground contact duration 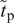 , an effective pendulum inertia 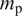 and a fixed damping ratio of 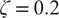 . Per estimate, 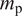 is chosen equal to the trunk mass 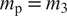 , and 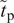 is estimated to be half that of 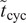 .

The pendulum’s rest length 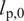 and floor coordinate 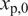 are chosen with an aim to produce a symmetric gait. For this purpose, 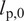 is chosen equal to the total length of the residual leg: 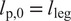 . 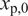 is set at the transition to HeStance to achieve rest length at impact: 
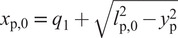
 . This transition is triggered when 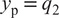 falls below a threshold height 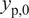 , which is chosen to correspond to the anticipated trunk height at heel strike of the residual leg, see Figure 2. The SLIP’s corresponding angle of attack can be calculated as 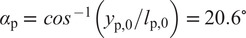 .

### 4.3. Walking: additional trunk support forces

In addition to forces resulting from the SLIP model, support forces can be applied to the trunk, which can have different functions. The application of any such force is optional. However, it is necessary to include a forward force to enable a gait that is energetically symmetric, since the SLIP model itself is purely dissipative, for which it can only generate asymmetric (though plausible) gaits without the injection of positive power.

In this work, we introduce a forward thrust force, which for purpose of simplicity is constant throughout stance of the intact leg (i.e., during activation of the SLIP model), but in addition, it is adjusted from step to step to regulate for the intended ambulation velocity 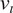 , as further explained below. Note that whereas this force is constant, the total horizontal force on the trunk is not, because it includes also the horizontal force components that is imposed by the SLIP model. It is not claimed that the resulting force profile is exactly similar to that exerted by a human, but it imposes possible and even realistic (due to the balancing of power injection) dynamics on the lower limb prosthesis, which is what matters for its functionality and feedback control.

In addition to the forward thrust force, other forces can be applied to model any type of realistic disturbance, caused by the human or environment, which could either help or perturb the prosthesis system and its controller. One example of common user behavior is lifting the hip of the residual leg to create additional ground clearance, enforcing an asymmetric gait. Especially for low ambulation velocities, it was found that the corresponding low inertial forces do not allow for sufficient knee flexion to prevent scuffing or tripping when using a passive knee and ankle prosthesis. Since the SLIP model parameters are defined to realize a symmetric gait, a vertical support force can be added to create additional hip lift. Note that whereas such asymmetric user behaviors should not be promoted, the prosthesis should also work for them. The possible implementation of a conceptual lifting force to enable walking with lower ambulation velocities is further explained below.

Also other forces or impulses can be applied, such as those resulting from a push, gust of wind or uneven terrain. These are not further investigated in this work, but are valid sources as disturbances for improving the robustness of a controller.

#### Horizontal thrust force for power injection and speed management

A support forward thrust force is applied to inject positive power and to regulate the intended ambulation velocity 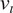 . The applied thrust force is constant throughout the stance phase of the intact leg and applied using the quadratic relation 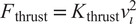 with gain 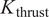 . This relation is used because the losses it aims to compensate for are strongly positively correlated with velocity, typically approximately quadratic or of higher order, depending on many factors of the non-linear system, including the employed controller, internal friction and impact conditions. Since 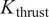 is not algebraically derivable and poorly predictable, it is updated once every step cycle at initiation of the stance phase of the residual leg, with the aim to converge to the desired ambulation velocity 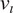 , requiring only an initial estimate (chosen as 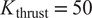 ). The update is based on an energy balance that compares the difference of the translational kinetic energy of the system for the actual and target ambulation velocity 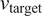 and a prediction of work done by the thrust force given an estimation of stride length:
(2)

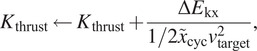

where 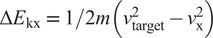 denotes the difference of translational kinetic energy for the measured ambulation velocity 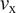 and target velocity 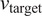 . 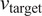 is defined as the average of the intended ambulation velocity 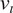 registered at initiation of the current and previous step cycle of the residual leg, as an implementation of a finite impulse response filter to prevent updates of 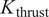 to overshoot. 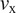 is calculated as the average velocity of the previous step cycle of the residual leg: 
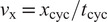
 , with 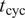 the duration between the last two heel strikes of the residual leg.

In addition, when the target velocity changes, an additional thrust force is added to accelerate or brake the system according to a prediction of the work required to realize a corresponding change of translational kinetic energy, employing a similar energy balance as that used for updating 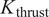 as in equation 2.

#### Vertical lift to emulate an asymmetric gait and prevent foot scuff

A support vertical lift force can be applied to realize additional lift of the hip during stance of the residual leg for lower ambulation velocities, to prevent tripping with a passive prosthesis. In the presented model, this vertical support force 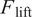 is synchronized with the SLIP rotation and defined as follows:

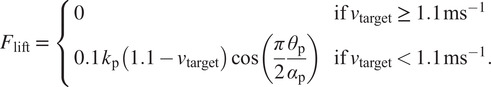

This implementation lifts the leg up to approximately an additional 1 cm for every 0.1 ms 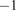 that the target velocity is lower than 1.1 ms 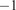 , which was heuristically found to avoid scuffing issues for ambulation speeds of at least 0.8 ms 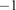 for the reference passive prosthetic leg that is showcased in Section 5. Additional lift is added during gait termination to prevent tripping. Naturally, depending on the goal of the simulation study, the implementation of an intentional asymmetric gait through a lift force can be increased, decreased, changed or removed entirely.

#### Other states

No force is applied to the trunk during swing of the healthy leg (HeSwing). Instead, While standing on both legs (Stand), half the system’s weight is applied vertically ( 
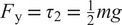
 ), and a horizontal damping force is applied to brake the system when terminating gait ( 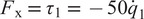 ).

## 5. Case studies

The framework is showcased with two case studies, accompanied by a proposal for a manual tuning procedure. The focus of this work is to present the framework as a general tool for prosthesis design, rather than to present a specific new prosthesis hardware or controller. As such, the first and main case study adopts an embodiment from a publicly available quasi-passive (variable damping) hydraulic commercial knee prosthesis design, which has already proven itself in the industry. Quasi-passive here refers to energetic passivity (dissipation), but to the ability to actively regulate the amount of damping (resistance) or braking force through an onboard microcontroller. This variable damping can be realized by using mechanical control valves (Auberger et al., 2016) or a magnetorheological fluid (Bisbee III et al., 2016).

Since the exact prosthesis hardware is not known nor available for verification, a second case study is introduced, which regards an internally developed powered knee prosthesis (Guercini et al., 2022), for which an initial version of the controller has been developed with the presented simulation framework. For purpose of demonstration, a passive spring-damper ankle prosthesis is implemented in both case studies. However, the framework is not limited to the use of passive ankles, and can also be used to incorporate a feedback control for active ankles.

Referring to Figure 1, the implementation of a prosthesis requires at least both the prosthesis controller and the prosthesis device modules to be defined, as they are specific to the prosthesis. In addition, the prosthesis sensors module can be defined to obtain a more realistic control, and dynamic and kinematic parameters of the dynamic model ( 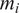 , 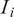 and 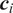 , and shank and foot dimensions) should be defined to correspond to that of the prosthesis. Other than these parameters, the human controller, hybrid dynamics engine and compliant ground model are not specific to the prosthesis.

### 5.1. Tuning procedure

Following a preliminary implementation of the prosthesis device and controller, initial parameter estimates can mostly be interpreted from reference material. Tuning of parameters or further refinements of the control system are done directly in forward dynamic simulations, which in our work are performed in Matlab Simulink (version 2020b). For new designs, the primary objective of a tuning procedure is to realize a feasible and stable gait with the closed-loop system. Secondarily, the objective is to improve (bio)mechanical characteristics for that gait, such as reducing internal peak forces, or to improve its robustness to deviations from the gait, such as different and changing ambulation speeds, starting and stopping behavior, but also other possible sources of disturbances and changes of human actions.

Generally, the holistic design and tuning of a multidimensional and intrinsically unstable forward dynamic system is a daunting task, for which typically is resorted to automated optimization techniques rather than manual heuristic processes. Especially for more complex systems like OpenSim, this is a sensible approach. However, disadvantages of such holistic optimization problems are that they are difficult to define and time consuming, and they provide lack of insight into the system. An initial heuristic approach can be favorable to set the scope of design solutions and possible future optimizations, for as long as the system is comprehensible. We found that a major advantage of the presented simplistic framework is that achieving a feasible solution (stabilization) and basic improvements can be achieved heuristically with a comprehensible and straightforward tuning approach. In addition, a simulation lasts on the order of only seconds,^2^ allowing for fast progression.

Whereas tuning remains an iterative process, the general approach is to move from the device to the controller, and from simple actions (slow walk) to extended behaviors (change of speed, starting and stopping, disturbances, etc.). In particular, the approach is to first obtain a feasible result for a stable unperturbed walking gait with a slow target ambulation speed, yet one that is fast enough to allow for passive knee flexion as a result of inertial forces. For this purpose, we found 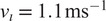 to be a good target velocity. Initial conditions of state variables for a cyclic (rather than starting or stopping) step are chosen as 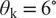 , 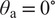 , the hip and trunk angles equal to their initial angle of their rough references for a walk cycle (see Figure 4, that is, 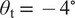 and 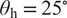 ), the trunk height so that ground contact with the heel is realized, and the trunk forward velocity so that it is 20% higher than 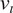 (a higher than average velocity prior to impact of the heel strike of the residual leg). Other initial state variables are zero.

**Figure 5.**
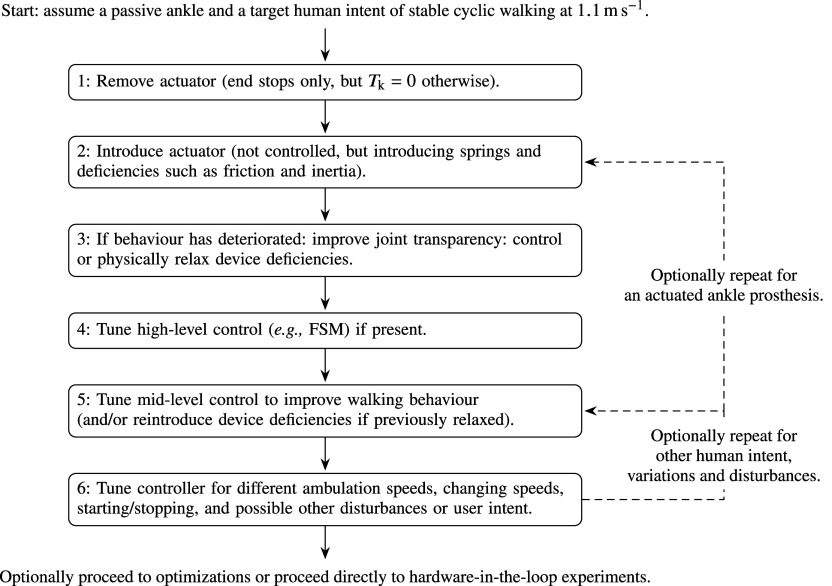
A proposed tuning procedure: whereas one could resort to optimizations to holistically tune the dynamic human-prosthesis system, the relative simplicity of the system also allows for manual tuning.

An overview of the proposed tuning procedure is visualized in Figure 5. To reduce system complexity, it is advisable to firstly assume the use of a passive ankle prosthesis, like in the presented case studies. For the knee prosthesis, the procedure can then be summarized as follows:

1. Remove the actuator(s) from the joint,^3^ leaving only the physical end stops but an otherwise transparent knee joint, and observe limit cycle instability for cyclic walking at 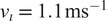 . The overly transparent knee joint typically results in a fall after several steps, but not immediately.
2. Re-introduce the actuator (friction, inertia, springs), and observe if cyclic behavior has improved or deteriorated. A minimum amount of friction (energy dissipation) generally improves the behavior, leading to a later fall or even a stable limit cycle.^4^ If instead behavior deteriorates, likely sources of friction and inertia are too high, leading to scuffing or tripping during swing as a result of insufficient knee flexion.
3. If the behavior has deteriorated, either directly attempt the implementation of a feasible controller that can counter some of these actuator or device deficiencies (e.g., friction, inertia) (step 5), such as a transparency, minimum jerk trajectory or impedance controller if the prosthesis is powered, or firstly relax device deficiencies until walking behavior is improved. Depending on the scope of the design study, these relaxations can be the foundation of an actual design iteration if the mechanical design has not yet been decided on. Otherwise, these changes can be temporal, and be reversed in a subsequent tuning stage when the controller is introduced.
4. If a high level walking controller is employed, such as an FSM, verify that it functions correctly, despite the lack of controller outputs. In case of an FSM, improve transitions where needed by analyzing sensor data, until it cycles through walking states according to expectations, without error.
5. Search to improve walking behavior by including and tuning controller outputs, and possibly by changing device parameters if the stage of design permits this (e.g., changing a spring constant). If device deficiencies had previously been relaxed, they can be reintroduced and compensated for by controller actions. (Bio)mechanical features can be used and pursued as reference for improving walking behavior (Grimmer and Seyfarth, 2014), such as:
  - A maximum knee flexion angle of approximately 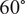 during swing. A significantly lower angle can lead to insufficient ground clearance, but an angle that is significantly higher might cause the knee to extend insufficiently when entering stance. Ground clearance itself can be monitored as *y*-coordinates of foot contact points, to understand if the model is (close to) scuffing or tripping.
  - Limited knee flexion during stance. In particular, at ground impact and lift-off, the knee is ideally nearly extended, and during mid-stance the knee flexes maximally approximately 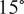 . Additional flexion increases the likelihood of buckling, especially when braking or terminating gait. Additional stance flexion resistance could be introduced to lower the maximum knee angle.
  - Reduction of internal peak forces as a result of impacts with physical end stops. For example, (angle-based) leg resistance during swing extension can be increased to lower impact forces with the knee extension end stop.
  - Reduction of prosthesis energy consumption and motor saturation. This is of particular interest for powered prostheses.
6. Once a satisfactory walking behavior is established for an ambulation speed of 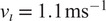 , the system can be challenged and tuned for different and changing intended ambulation speeds and gait initiation and termination, which can be seen as disturbances to the control system. Especially braking challenges the controller during stance, as it results in additional flexion load on the knee that could result in the system buckling to the ground. At this stage, also high level intent recognition and speed adaptation control modules can be experimented with.

The successful completion of these steps results in a prosthesis and controller system that is feasible for a certain repertoire of possible human behaviors, which formulate a strong basis for design realization. However, it should be noted that the converged solution is likely not unique, nor is it claimed to be optimal in any sense, especially because the human system is simplified, and not exercising optimal actions itself.

If the design project also includes the development of an active ankle prosthesis, one could proceed to its introduction and repeat the tuning process. In addition, it is certainly possible to repeat the tuning process for a broader range of disturbances and human actions (e.g., change or removal of support forces), trajectories (e.g., different hip reference trajectory) and parameters (e.g., different body sizing), in order to improve controller robustness for a broader repertoire of possible human behaviors. However, it should be noted that the effectiveness of manual tuning might crawl to a slow when increasing the dimensionality of human deviations, and that it might instead be more productive to implement an optimization procedure at this stage.

### 5.2. Variable damping commercial leg

**Figure 6.**
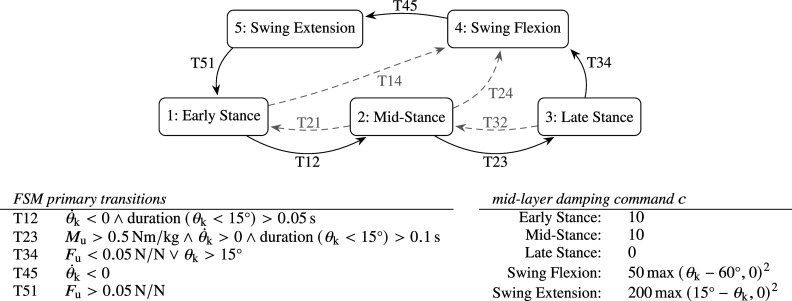
Prosthesis FSM and state-based variable damping control for walking (states 1 to 5) and standing (states 1 and 2), largely inspired from Bisbee III et al. (2016), implemented for proofing the simulation framework. Dashed transitions incorporate standing, non-standard walking and corrective behavior, which are not triggered in this demonstration.

The implementation of the commercial leg is inspired from a simplification and interpretation of material presented in a patent of Össur (Bisbee III et al., 2016). Its FSM and state-dependent damping formulae are depicted in Figure 6. Parameter choices of the FSM follow largely from direct interpretations, and are partly tuned according to the procedure described in Section 5.1, as further detailed in the subsequent Section 5.2.2.

The controller runs at a frequency of 200 Hz,^5^ and the encoder is quantized for a resolution of 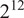 ticks per revolution, as realized by the prosthesis sensors module. The controller commands the quadratic damping coefficient 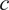 , which for purposes of demonstration is assumed to be a perfect damping source, so that the applied torque by the damper on the knee equals 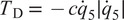 , as implemented by the prosthesis device module. This module also implements sources of friction to define the knee torque, and implements a passive ankle prosthesis to define the ankle torque, as further detailed below. Kinematic and dynamic parameters of the prosthesis are according to those listed in Table 1.

#### 5.2.1. Prosthesis device

In addition to the damper torque 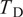 , a realistic amount of joint viscous friction, stiction, soft parallel extension spring and an extension end stop has been added to the prosthesis device model, so that

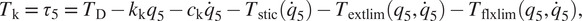

with stiffness 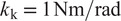 , damping 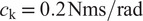 , stiction 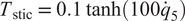 and extension and flexion end stop torques 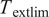 and 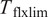 , respectively.

To accurately incorporate the behavior of an end stop of finite thickness, it is implemented using an explosively progressive non-linear compression spring element. For this purpose, the one-dimensional Neo-Hookean element 
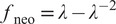
 is employed, where 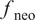 is proportional to the output force and 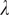 the extension ratio of the compressed element, which accurately describes the behavior of materials with a Poisson ratio near 0.5, such as rubbers (Treloar, 1975). By including also a damping term, the extension limit torque 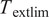 is computed as follows:

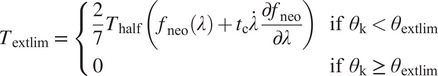

with the extension ratio 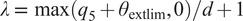 , starting angle 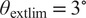 , thickness 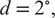 torque at half compression (i.e., 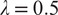 , or 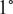 of compression) 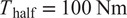 and damping time constant 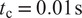 . An identical end stop has been implemented for flexion, which operates in the opposite direction at a starting angle of 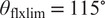 , producing 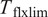 .

The prosthesis system also includes the ankle, which is modelled to be passive, and as such does not require a controller. It is implemented as a linear spring-damper system: 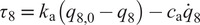 , with rest angle 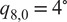 , stiffness 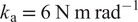 and damping 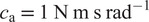 .

#### 5.2.2. Results

##### Intermediate results

**Figure 7.**
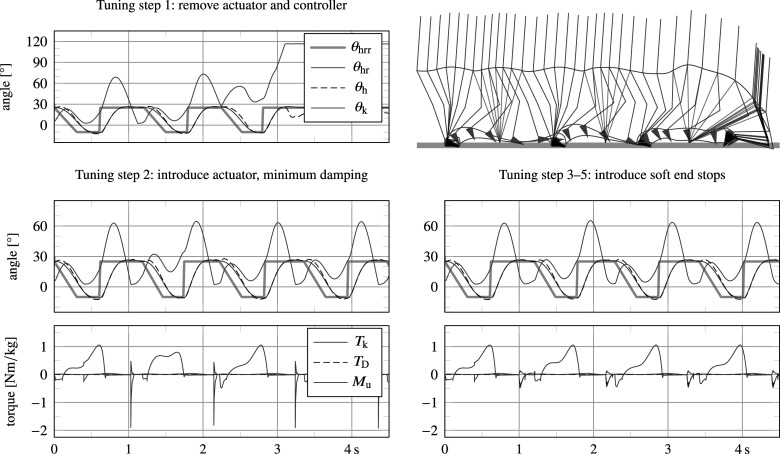
Intermediate results of the tuning procedure, showing angular trajectories of the knee 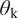 and hip 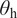 (including also the hip rough and smooth reference trajectories 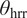 and 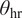 , respectively), and torque values of the knee 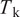 , damper 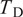 and tube sensor 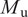 . The top graphs show instability as a result of removing the actuator (both its friction and spring and controller output). By introducing the actuator without any controlled output, a limit cycle gait can already be achieved (step 2), but it is subject to internal impacts and sensitive to disturbance. The introduction of angle-based damping (soft end stops) rids these internal impacts and improves stability of the gait cycle (step 3).

The procedure described in Section 5.1 has been used as a general guide to tune a number of controller parameters. Figure 7 shows several of the intermediate results of this process. The top graphs depict results for walking with 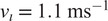 and setting all joint friction and stiffness values and mid-layer controller parameters to zero, leaving only the end stops to generate a knee torque, so that 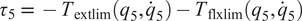 . The system ends up falling due to an overly transparent knee joint, but still manages to complete several steps.

The bottom left graphs show results for reintroduction of basic joint damping and stiffness values (i.e., 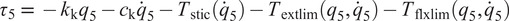 ). The introduction of minimal damping caused the system to no longer fall, but neither did it converge to a single-step limit cycle, possibly due to the lack of angle-based damping (soft end stop) in swing flexion. In addition, the knee joint collided with the physical extension end stop at the end of swing extension due to the lack of damping, resulting in high impacts, which are visible in the bottom left figure. As regards for the high level controller, we found that the FSM—directly interpreted from Bisbee III et al. (2016)—correctly identified transitions, except for occasional preliminary transitions to late stance (pre-swing). This was caused because of a different moment reading. Where Bisbee III et al. (2016) uses a moment measure closer to the knee joint to detect a transition to late stance with a threshold of 0.2 N m kg 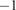 , we employ a moment measure closer to the ankle (i.e., the tube sensor), which is higher than 0.2 N m kg 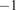 during most of stance. To resolve this, we have increased the threshold to 0.5 N m kg 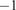 , as listed in Figure 6. The framework has proven exceptionally useful to understand sensor data that are customary to specific design solutions, of which little or no data are available in the literature.

The bottom right graphs in Figure 7 show results after introduction of angle-based mid-layer damping commands in swing flexion and swing extension (soft end stops), as listed in Figure 6, resulting in a more stable gait and no more knee joint impacts with the physical knee extension end stop. However, due to the lack of stance support, the model was still found to exhibit high knee stance flexion angles, and to fall down when lowering the ambulation speed. This was resolved by introducing and tuning additional damping commands in stance, as listed in Figure 6.

##### Main results

**Figure 8.**
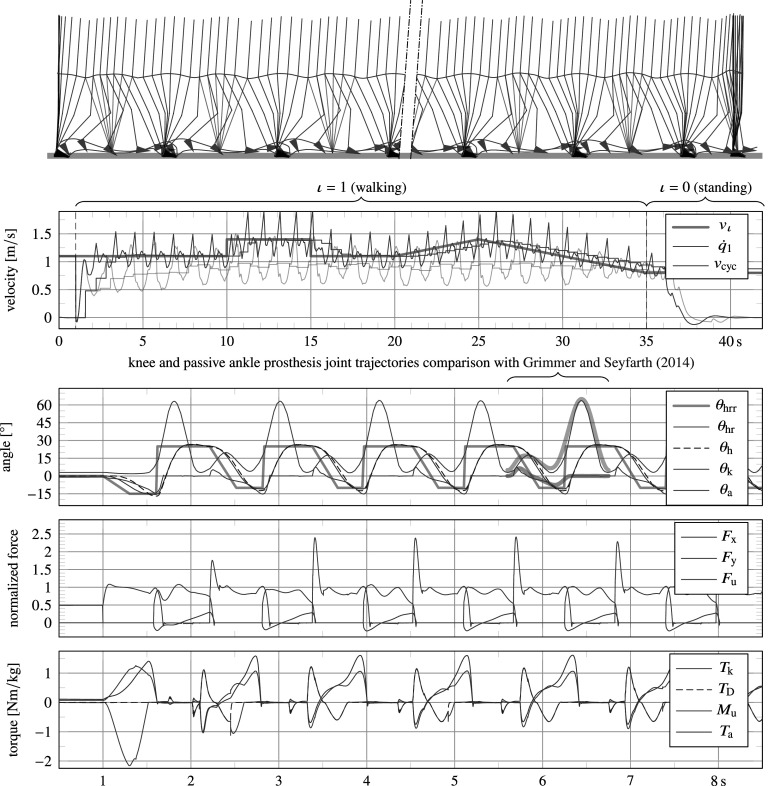
Simulation results of a walking trial, starting from and ending in standstill. The first plot shows animation frames, sampled at 10 Hz, with the SLIP drawn in red, and where the hip, toe and heel trajectories are drawn to demonstrate gait symmetry and foot clearance. The second plot shows the ambulation velocity 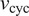 (updated at heel strike of both the intact and residual leg) of the full simulation, which shows that it correctly tracks the intended velocity 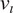 with a delay of several steps. In this graph, transparent lines correspond to results of the simulation for complete removal of the forward thrust force, leading to an asymmetric “limping” gait and poorly tracked intended ambulation speed. The last three plots show various joint positions (global hip angle 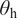 , its rough and smooth reference trajectories 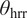 and 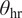 , knee angle 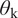 , and ankle angle 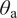 ), forces (horizontal 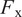 and vertical trunk force 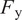 and tube force 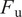 ), and torques (knee torque 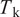 , of which torque contributed by the damper 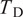 , tube moment 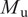 and ankle torque 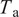 ), respectively, for the first 8 s of the simulation.

All final controller parameter values are depicted in Figure 6. Simulations have demonstrated stable walking for a range of ambulation velocities of at least 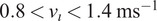 . Figure 8 shows results from a 40 s trial, of which various joint angles, forces and torques are plotted for the first 8 s. During the 40 s trial, the system starts from a vertical standing orientation, is commanded to start walking ( 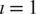 ) at 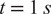 with an ambulation velocity of 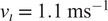 , to accelerate and decelerate with both step and ramp commands, up to 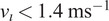 and down to 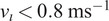 , and to stop walking ( 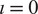 ) at 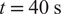 .

The results plotted in Figure 8 show high resemblance with human data recordings found in literature. The knee trajectory shows a healthy stance bounce and peak flexion values similar to those found in healthy user data, a comparison of which is shown in Figure 8 with data from Grimmer and Seyfarth (2014). The ankle trajectory instead is very similar specifically to that of users wearing a passive ankle prosthesis, a comparison of which too is shown in Figure 8 with data from Grimmer and Seyfarth (2014). In addition to kinematic data, also kinetic data follow expected patterns. Notable is the observed ‘camel back’ shape of the vertical force. This shape is also present in the tube force 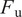 , but it appears with additional bounces since the feet have been modelled with only two ground contact points. In addition, it is sensitive to peaking at impact due to the high rigidity of the rigid-body system. Also peak values of torque measures correspond well to those found in literature, namely ca. 1 N m kg 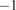 and 1.5 N m kg 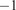 for the knee and ankle torque, respectively, at an ambulation velocity of 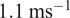 .

##### Symmetry

The SLIP model is observed to successfully generate a symmetric gait regarding hip height, except for low ambulation velocities ( 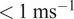 ), for which we have intentionally introduced an asymmetric lift force (Section 4.3) to generate sufficient ground clearance. Nevertheless, the asymmetry of the hip motion is minimal and barely visible in the animation plot of Figure 8. While walking with an ambulation speed of 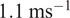 (i.e., when the lift force is not yet active), the hip peaks at a height of 902 mm and 900 mm from the ground for the stance phase of the residual and intact limb, respectively. This height corresponds with the leg length 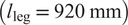 in combination with ground softness and minimal knee flexion. In the last step prior to terminating the walking gait, the hip peaks at a height of 911 mm during stance of the residual leg as a result of the lift force, which prevents it from falling. If instead the lift force is entirely removed from the simulation, the model is observed to start scuffing around 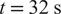 , when decelerating to an ambulation speed of approximately 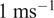 , and falls two steps later, before finishing the trial.

Regarding symmetry of forward velocity, it can be observed that 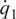 oscillates slightly more during stance of the intact (SLIP) limb than during that of the residual limb. Such an asymmetry is to be expected given the different modeling approach for the residual and intact leg: one having inertia, the other being a mass-less SLIP. However, the average stance phase (ignoring initiation and termination steps) of the residual and intact leg last 53% and 57% of the stride cycle, respectively (i.e., the double support phases amount to 10% of the stride cycle). This is only a small difference, which falls within the range of asymmetries found in recorded data (Grimmer and Seyfarth, 2014). The forward thrust force (Section 4.3) during stance of the intact limb allows for this near-symmetry, as it enables power injection during both stance phases. Instead, when this thrust force is completely removed, the only positive power injection occurs during stance phase of the residual leg. As a result, the stance phase of the intact leg lasts longer, and the gait becomes reminiscent of limping. In fact, by doing so, the average stance phase of the residual and intact leg were measured to last 65% and 45% of the stride cycle, respectively. Interestingly, despite this strong asymmetry, the model did not fall. However, the intended ambulation speed was neither tracked properly, as this was also regulated by the thrust force. The resulting ambulation speed and 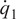 of this simulation are also shown in the velocity plot of Figure 8, in transparent colors.

#### 5.2.3. Introducing socket dynamics

As mentioned in Section 3, it is possible to introduce additional joints and bodies to pursue more realistic results, at the cost of increased simulation time. One such addition concerns the interaction between the patient’s stump and socket, which is currently modelled to be rigid, given the utilization of a single rigid body to model both (i.e., the thigh), but in reality is reported to be significantly soft (LaPrè et al., 2018). Prosthesis dynamics and control mostly act in the sagittal plane, for which the most relevant degrees of freedom are pistoning (longitudinal or vertical displacement) and anterior–posterior tilt (LaPrè et al., 2018). Instead, anterior–posterior displacement (forward horizontal displacement) is less relevant.

Two joints and bodies have been introduced between the hip and knee joints to model stump-socket softness for vertical displacement and anterior–posterior tilt, respectively. As a result, the dynamic system now consists of 10 instead of 8 joints: the stump-socket joints are joints 5 and 6, and the knee, tube, and ankle joints have moved down in the serial kinematic chain from joints 5 to 8 to joints 7 to 10. Per demonstration, The location of the two new joints are exactly halfway the thigh, and inertial properties of bodies have not been altered (the corresponding two new bodies have no mass). They are furthermore configured as forward dynamics joints, and per demonstration, their softness is modelled with translational and rotational linear spring-damper systems, so that

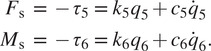



Whereas exact stiffness and damping gains are unknown and patient specific, here stiffness gains are chosen to realize any displacement difference on the order of 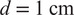 , in line with calculated displacement errors of markers in inverse kinematic studies (LaPrè et al., 2018). Damping gains are chosen to realize a system that is at least critically damped. As such, for the longitudinal displacement, we have selected the translational stiffness as 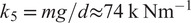 and the translational damping as 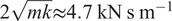 . For the rotational joint, we have selected 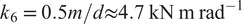 , to result in approximately 0.01 rad of displacement, and 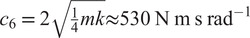 , where 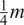 is an upper estimate of effective inertia at the joint.

Without applying any further adjustments to the system, the controller or human intent, the simulation has been rerun. Generally, odds for instability are higher with increased softness, especially when the prosthesis and human controller are not further refined. However, results show that the model did not fall for the full duration of the 40 s trial. The corresponding simulation time has approximately doubled, but was still under 1 min. Nevertheless, when rerunning the simulation for stiffness values half that of the originally selected ones, a fall is observed around 11 s, after the increase of ambulation speed.

**Figure 9.**
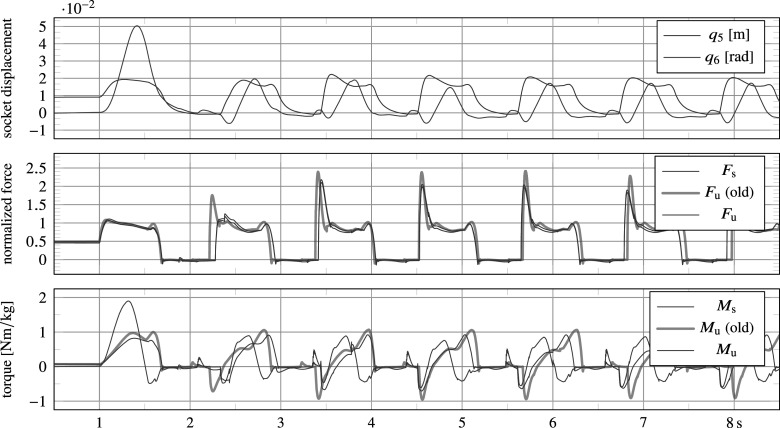
Results demonstrating the effect of socket dynamics on the system, using otherwise similar properties and inputs as that of the simulation whose results are shown in Figure 7, for purposes of comparison.

Results of socket displacement, forces and torques of the first 8 s for the originally selected stiffness values are shown in Figure 9. For comparison with previous results, the tube force and torque in Figure 8 have been plotted too. As expected, the force is very similar, but the moment is different. Nevertheless, the force signal is visibly smoother and its peak values have reduced with up to 20%, as a result of the new softness in the system.

### 5.3. Powered research leg

**Figure 10.**
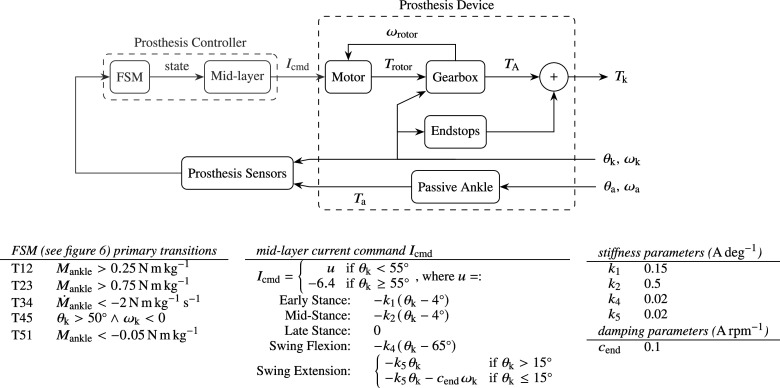
Overview of the FSM-impedance-based prosthesis controller module and electrically actuated research prosthesis device module, as designed and tuned to enable walking, starting and stopping. The FSM states are the same as those in Figure 6.

The second case study is that of an existing powered research prosthesis (Guercini et al., 2022). In particular, this research leg employs a dual motor architecture to assist multiple types of activities. It has a small, fast, yet low torque actuator, which is permanently engaged with the knee joint. In addition, it has a large, slow, but high torque actuator, which can optionally engage to provide additional torque in extension for activities that benefit from this, such as stair climbing. The use of only its small actuator should suffice to realize walking, despite its rated peak torque limit of approximately 30 Nm, whereas the knee torque of healthy subjects is observed to peak at approximately 1 NN 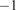 during walking (75 Nm for a subject of 75 kg). The presented simulation framework can be used to demonstrate this. Toward this purpose, an FSM-based controller inspired from that presented in Section 5.2 has been created, as shown in Figure 10. This controller has been used as the basis for the control system that has been presented for experimental validation by Guercini et al. (2022), as realized with a Speedgoat interface in combination with a subject wearing an able-bodied adapter.

The prosthesis device is mainly different from that of the commercial quasi-passive hydraulic knee prosthesis (Section 5.2) in that the simplified hydraulic damping actuation model has been replaced for an electric drive train, featuring a model of a current-controlled DC motor and the gearing, which takes the input of a commanded current rather than a damping coefficient, as shown in Figure 10. The purpose of this implementation is to incorporate (1) realistic effects and limitations as a result of gearbox friction, fast shaft inertia and supply voltage and current limitations, (2) the possibility to analyze efficiency and heating as a result of joule and frictional losses. The end stops described in Section 5.2 is also included in the system of the research prosthesis.

To reduce simulation time, high-frequency dynamics are avoided by modeling the field-oriented controlled brushless Maxon EC 45 flat 24 V motor in continuous time and as a perfect current-commanded but saturated torque source that takes supply voltage and current limitations into account according to the equivalent brushed DC-motor model. With a torque constant of 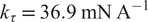 , a terminal resistance of 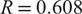 , a maximum permissible current of 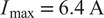 (ca. double that of the specified nominal current) and a supply voltage of 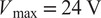 , the theoretical maximum torque and no-load speed are 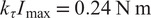 and 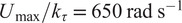 _,_ respectively, on the fast shaft. The gearbox model incorporates drive-direction dependent (forward or backward) stiction, viscous friction and coulomb friction of the Conedrive CBC140100 strain wave gear with a gear ratio of 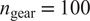 , and incorporates inertia of both itself and the motor rotor. The gearbox-motor unit connects to the joint via a relatively stiff linear spring element with a stiffness of 1000 N m rad 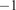 and a damping of 30 N m s rad 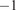 , to account for gearbox and joint flexibility. The knee torque 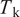 is computed as the sum of the output torque of the gearbox 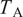 and the end stop torque 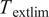 .

#### 5.3.1. Results

At the time of controller development for the work by Guercini et al. (2022), the focus was on realizing a single feasible walking gait for experimentation with an able-bodied user, without taking other actions into account or prosthesis energy consumption.

**Figure 11.**
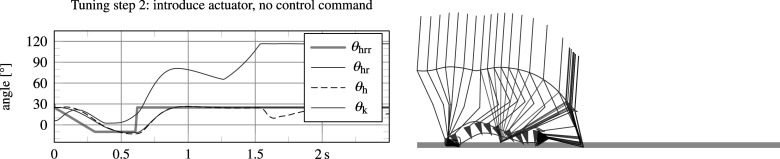
In contrast to the variable damping leg, the powered research leg shows impeded walking behavior when its actuator is introduced without a control output.

Following the procedure in Section 5.1, a main difference between the variable damper knee and powered knee is that inclusion of the electric actuator without any control output (step 2) leads to deterioration rather than improvement of walking behavior: not being able to complete a single step cycle, as shown in Figure 11. The presence of inertia and significantly more friction of the electric actuator cause the knee to extend too late and slow during swing, causing the system to trip. A fixed extension torque has been applied for knee angles 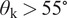 to reliably overcome gearbox stiction and realize a repeatable transition from swing flexion to swing extension.

In addition, an impedance controller has been introduced that includes non-zero stiffness gains. The purpose in swing is countering friction to accelerate knee flexion and extension, especially at initiation of the walking gait, for the system would otherwise trip and fall. In stance, the purpose of these terms is providing additional support for braking. The introduction of additional damping gains was not necessary, as the gearbox friction already realizes sufficient damping on its own. Nevertheless, similar to the previous case study, damping has been introduced to brake the knee near extension ( 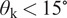 ) in swing extension, to lower the impact torque with the extension end stop.

**Figure 12.**
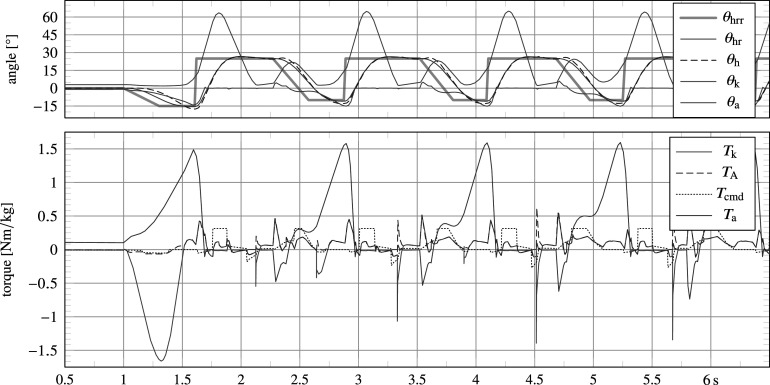
Simulation results of an electrically powered research prosthesis, which demonstrate the possibility of realizing a walking gait including a knee bounce with a commanded knee torque 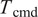 that has a peak value (ca. 24 Nm) significantly lower than that found in a biological knee (ca. 75 Nm for a person weighing 75 kg). Due to gearbox friction and inertia, the actuator output torque 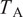 is significantly different from the commanded 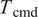 . Spikes correspond to collisions with the knee extension end stop.

Results of the simulation for walking at a target velocity of 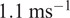 starting from standstill are shown in Figure 12, corresponding to the designed and tuned controller shown in Figure 10. In this figure, 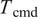 corresponds to the commanded current 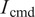 , saturated by its absolute maximum of 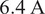 , multiplied by the torque constant 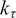 and gear ratio 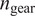 , and as such represents a hypothetical or ideal torque output considering a drive train that is free of friction and inertia, which peaks at ca. 24 Nm. Impact torques with the extension end stop are still visible (e.g., at 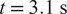 , 4.3 s, 5.6 s, etc.), but have been reduced by approximately 40% due to the inclusion of additional damping in swing extension.

Foremost, the results illustrate the possibility to realize a possible walking gait despite the use of a weak actuator with limited torque output. Due to friction, the actual torque output 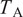 is lower when forward driven, but higher when backward driven, or during rapid accelerations as caused by drive train inertia, for which it is significantly different from 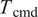 . The latter is visible during the aforementioned collisions with the extension end stop, which—in combination with the relatively stiff drive train—result in a high reaction torque on the actuator ( 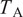 ). This provides an argument against the use of stiff (non-series elastic actuated) electric actuation with high inertia in knee prostheses.

An analysis of energy flows of the actuator can be performed to illustrate the sub-optimal performance of the present hardware and control solution. These can be calculated by integrating the mechanical and electrical power flows from its various components over time, which is preferably done for a whole number of steps. Average power can then be calculated by dividing the resulting energy figures by the total duration 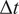 of these steps. The actuator was shown to damp the system by doing negative work, calculated as 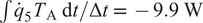 , averaging five whole steps from the third step onward, from 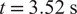 to 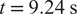 ( 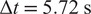 ). Yet, the motor provides a net mechanical power of 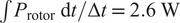 to the actuator, where the motor rotor mechanical power 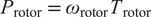 , with 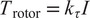 the applied torque on the motor rotor (fast shaft), and 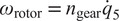 its rotational velocity. This suggests 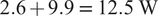 of heat losses in the transmission alone. Nevertheless, the power flows in the motor are significantly higher than the net average figure of 2.6 W. In particular, it is the sum of positive work of 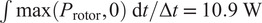 and negative work of 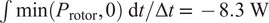 . However, the main issue is with electric energy consumption, calculated as 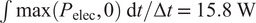 , of which up to 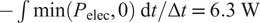 is possibly eligible for regeneration,^6^ where the electrical supply power 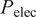 is estimated according to the DC-motor equivalent 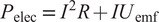 , with the counter-electromotive force 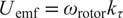 , and 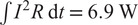 is permanently lost to joule heating in the windings of the motor. Depending on energy regenerative capabilities of the electronics, this suggests an efficiency for the motor alone of only 16% to 27%. Other control strategies can likely improve this figure. Nevertheless, also regarding hardware, it is noteworthy that—despite the specific design choice of using two motors of which only the smaller and faster one is used for passive activities such as walking—it still suffers from high inertial reaction torques and friction for which the motor partly needs to compensate, in order to realize a (transparent) walking gait. An optimization of not only the controller, but also the hardware—featuring a faster and more efficient drive train still—could lead to significant reduction of energy consumption.

The simulation provides a basis for an initial feasible control scheme to perform walking experiments of the research leg (Guercini et al., 2022). However, after its implementation, whereas most parameters are kept unchanged, changes to the controller are nonetheless made on the real device. After all, the presented model remains a simplification of reality, in particular regarding human behavior. Whereas the model produces feasible results for a certain scope of possible human behaviors, it is not guaranteed that the human will exhibit exactly those behaviors in reality, nor does the model take human psychology into account. Other reasons for controller changes include unknown or imperfectly modelled sources of friction or device imperfections, leading to the removal or inclusion of additional friction compensation terms to the implemented controller.

## 6. Discussion

The presented results of the case studies demonstrate that realistic closed-loop dynamic behavior can be generated using a reduced-order model of the human that emulates only a fraction of the degrees of freedom found in a real human and employs a control system that is substantially simpler than that of a real human. Due to the efficiency and choice of the implemented dynamics, the simulations run fast, allow for manual tuning and do not require optimizations to run, unlike more complex platforms such as OpenSim. The ability to tune a system manually and retrieving results quickly facilitate obtaining new insights into the system, which can aid in refining the scope of design solutions. This renders the platform useful as an offline design tool for new lower limb prostheses and their controllers, but also for investigating possible alterations of existing designs, such as those regarding the usability of compromised or new types of sensor signals or actuators. In general, whereas the human is modelled to be very simple, there is no constraint on modeling complexity of the prosthesis, which can help answer many different technical design questions that require a closed-loop control. For example, it can be investigated which range of filter cut-off frequencies, encoder resolutions or series elasticity in the drive train still produce acceptable results. Exemplary to the first case study in particular, the simulations have proven useful to interpret the sensor signals generated by the tube force and moment sensor, which do not directly correspond to biological knee or ankle data. The framework is also useful to investigate energy flows in the prosthesis, as illustrated in the second case study.^7^

Most of the dynamics imposed on the prosthesis show high correspondence with data from literature. Nevertheless, the human model can be further expanded to be more realistic, at the cost of simulation speed. For example, additional ground contact points could be introduced to more accurately emulate the foot shape and thus the ground reaction force, and additional joints can be added to allow for more flexibility and thus lower peak torques. An example is the inclusion of joints that represent the socket in the thigh, which has been showcased in the first case study. In addition, it could be considered to expand the model with also the intact limb, or upper limbs, but the main advantage of the SLIP model is its low dimensionality and ease of realizing stabilized gait, despite its passivity, requiring only an initiation reflex. Modeling also the other limb requires a significantly more complicated closed-loop human controller, which also complicates the tuning process, possibly stripping the feasibility of employing heuristic approaches.

However, whether the dynamics on the prosthesis are less or more realistic, a successful tuning only addresses a certain repertoire of possible human behaviors. Whereas this nevertheless formulates a strong basis for the realization of a design or controller implementation, it should be noted that the converged solution is likely not unique or optimal. It is never guaranteed that the human will exhibit exactly those behaviors for which the system is tuned in reality. It is certainly possible to expand the tuning process for additional human actions and variations, but the effectiveness of manual tuning might crawl to a slow when increasing this dimensionality. At this point, it might be more beneficial to implement an optimization procedure. A valid optimization target is the enhancement of closed-loop robustness for multiple behaviors, possibly in combination with reducing internal joint peak forces (or torques) or prosthesis energy consumption. However, unlike more complex musculoskeletal models, the presented framework is less suitable for optimizing a single gait for energy consumption of the human, due to the simplifications of the modelled human. Instead, prosthesis device and controller optimizations have not been explored in this work, but they are an interesting topic for future research. For example, a selection of prosthesis controller parameters could be optimized for maximizing a robustness measure such as the basin of attraction or gait sensitivity norm (Hobbelen and Wisse, 2007), using a selection of disturbances or changes of human control parameters.

Regarding behavioral expansion for either manual tuning and optimization processes, the human controller can be expanded to become less or more intelligent, and to include additional actions. In fact, the hybrid dynamic simulation framework has intentionally been designed to be easily configurable and extendable. For one, joints can individually be configured as either forward or inverse dynamics joints for different simulation. Secondly, the human controller is designed specifically to not be limited to exhibit limit cycle behavior. In particular, the ability to incorporate other activities has been facilitated in order to allow for the development or verification of higher level control, such as intent detection or ambulation speed estimation. This is realized by the presented two-step method of trajectory generation, allowing for generating smooth reference trajectories on the fly. In this work, the method is demonstrated by the inclusion of a standing behavior, its transitioning from and to walking and the ability to dynamically change the intended ambulation speed, but can be extended to include additional actions such as a deviating hip trajectory to emulate obstacle avoidance, or sitting and stair climbing, which is facilitated through the already present action of standing still. Correspondingly, some of these actions also require the dynamic model to be extended with additional contact models to include interactability with objects such as chairs and stairs. Lastly, some actions can benefit from configuring the trunk orientation as a forward, rather than inverse-dynamics joint, to implement torque limits of the human and to make the human controller more realistic.

## 7. Conclusion

This manuscript has presented an efficient holistic modeling and simulation framework for design and development of knee and ankle prostheses, featuring a reduced-order hybrid dynamic multi-rigid-body model of a human that models only the prosthetic limb and trunk. Whilst employing a simple SLIP-based walking controller for the human, we have successfully demonstrated the ability to accurately simulate non-limit cycle walking with two case studies, including starting and stopping behavior and dynamically changing the ambulation speed. The two case studies model a commercial variable damping hydraulic knee prosthesis and an electrically powered research knee prosthesis that employ well-established finite-state machine and impedance-based control strategies. Results were found to correspond well to those in literature, and the system has been designed to facilitate modification and expansion of human actions and prosthesis properties. The combination of high flexibility, accuracy, and simplicity make the proposed modeling and simulation framework a promising tool for designing and validating lower limb prosthesis controllers and mechanisms.

## Data Availability

The authors confirm that the data generated by this study are available within the article and its supplementary materials.

## Footnotes

1 Alternatively, if it is desired to model a degree of structural stiffness, these joints can be configured as forward-dynamics joints and modelled as stiff spring-damper systems, at the cost of simulation speed.

2 Despite being implemented on the relatively slow development platform Matlab Simulink, simulations have been completed in less time than the specified duration (stop time) of the simulations, using an outdated laptop from 2017 with the following CPU model: Intel(R) Core(TM) i7-7700HQ CPU 2.80GHz.

3 This includes removal of torques as a result of controller output as well as that caused by friction, springs and inertia, as defined in the prosthesis device module.

4 We found this behaviour to be in line with reality as well, where users were able to walk on a variable damping prosthesis that is switched off with minimum damping applied, albeit uncomfortably so due to the lack of stability during stance.

5 Note that, whereas the leg is energetically passive, the amount of damping is still actively regulated by an onboard microcontroller.

6 These numbers imply 100% efficiency of driver electronics and the battery recharging process.

7 To an extent, such studies can also be done with inverse dynamics simulations, but they are prone to placement of unnecessary constraints on trajectories that lead to unrealistic or unfavourable outcomes, especially on systems that exhibit series elasticity.

## Supplementary material

For supplementary material accompanying this paper visit https://doi.org/10.1017/wtc.2023.6. click here to view supplementary material
